# Probabilistic analysis and resistance factor calibration for deep foundation design using Monte Carlo simulation

**DOI:** 10.1016/j.heliyon.2018.e00727

**Published:** 2018-08-13

**Authors:** Thuy Vu, Erik Loehr, Douglas Smith

**Affiliations:** a Department of Civil Engineering, University of Texas Rio Grande Valley, 1201 W. University Dr., Edinburg, TX, 78539, USA; b Department of Civil Engineering, University of Missouri Columbia, 105 Jesse Hall, Columbia, MO, 65211, USA; c Department of Mechanical Engineering, Baylor University, Waco, TX, 76798, USA

**Keywords:** Civil engineering

## Abstract

The method of incorporating the sources of parameter uncertainty is crucial when conducting probabilistic analysis for service limit state (SLS) design of a deep foundation. This paper describes the method of using Monte Carlo simulation for probabilistic analyses and for calibration of resistance factors of drilled shafts at SLS. The paper presents discussions on the finding of an impossible case, where the different combinations of load, variability of soil strength and target probability of failure made it impossible to calibrate the SLS resistance factors. Resistance factors for drilled shafts in shale are introduced, and were found to be responsive to load levels. The higher load level, the lower the resistance factor. These findings help smooth the transition from allowable stress design to load and resistance factor design for geotechnical engineers.

## 1 Introduction

Geotechnical engineers have been working to transition from allowable stress design (or working stress design), which has been used for many years, to load and resistance factor design (LRFD). In allowable stress design, every input parameter is treated as deterministic, and the uncertainty in each design step is combined into one global factor called the “factor of safety.” In LRFD, a design starts with identifying all possible failure modes or limit states. The design reaches a limit state when a component of the structure does not fulfill its prescribed function. The LRFD limit states often are separated into ultimate limit state (ULS) and service limit state (SLS) categories. The ultimate limit state relates to geotechnical strength failures; for example when the applied load is equal to the resistance. The SLS is when a component of the structure deforms beyond a prescribed amount; for example when the vertical displacement of a drilled shaft is larger than the prescribed limiting settlement. In a general form, the performance function, denoted as *g,* is the difference between the nominal resistance *R* and the nominal load *Q* as in Eq. (1):

(1) g=R−Q

**Fig. 1:**
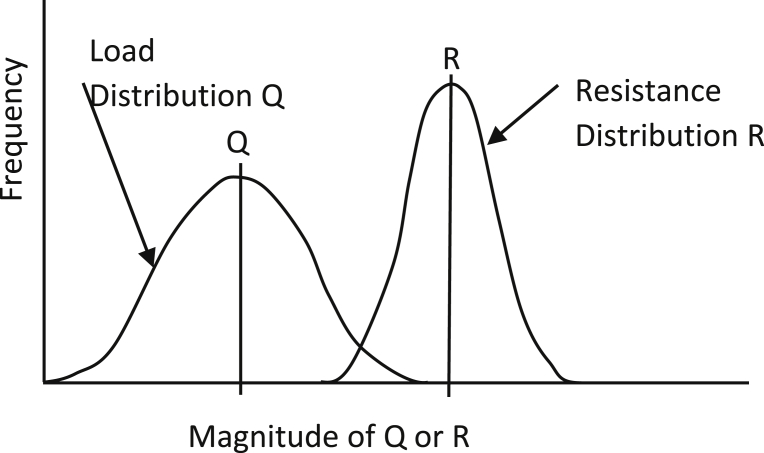
Frequency distribution of random values of load and resistance.

When the performance function *g* is equal to or less than zero, it defines an unsatisfactory performance region; however, if *g is* larger than zero, this indicates a satisfactory performance region. For probabilistic analyses, the resistance *R* and load *Q* are probabilistic parameters, each having its own distribution as shown in Fig. 1. The overlap area under the two curves in Fig. 1 is associated with the area of the failure region, which refers to the probability of failure for the design.

(adapted from Allen et al., 2005)

Methods to evaluate SLS for deep foundations have been proposed. Zhang and Chu (2009) proposed partial factors to satisfy serviceability limits for several different settlement prediction methods, with target reliability indices ranging from 1.0 to 2.5. The partial factors are roughly equivalent to resistance factors that range from 0.2 to 0.5; however, the partial factors are strictly only appropriate for use with nominal working loads equal to 50 percent of the ultimate foundation capacity. Resistance factors proposed by Misra and Roberts (2009) for establishing an allowable shaft capacity at the SLS range from approximately 0.25 to 0.55 for a target reliability index of 2.6. However, the resistance factors were found to depend on foundation length and diameter in addition to the variability of the soil-shaft interface resistance. Phoon et al. (1995) similarly proposed deformation factors (i.e., SLS resistance factors) for drilled shafts in medium, stiff, and very stiff clay with different coefficients of variation (COV) for undrained shear strength. The proposed factors ranged from 0.48 to 0.65, but are strictly appropriate for a target reliability index equal to 2.6.

Because of these constraints and challenges, current AASHTO LRFD provisions (AASHTO, 2014; Brown et al., 2010) adopt load and resistance factors of *unity* for SLS design. This position realistically reflects temporary adoption of historical design practices because of the current lack of practical methods for implementing probabilistically calibrated load or resistant factors for the SLS. This paper describes a proposed procedure that allows SLS design to be performed to achieve some desired target reliability without requiring case-specific calibration or more rigorous reliability-based design.

## 2 Background

Several probabilistic approaches are used in reliability-based design and in the LRFD resistance factor calibration. The most frequently used methods are the first-order second-moment (FOSM) method, the first-order reliability method (FORM), and the Monte Carlo simulation method. Details about the methods have been described in the literature (Ang and Tang, 2004; Baecher and Christian, 2003; Griffiths and Fenton, 2007; Harr, 1987). FOSM is based on a Taylor series expansion of a performance function (Baecher and Christian, 2003). FORM is the linear approximation of a limit state (Phoon et al., 2003; Phoon and Kulhawy, 2008), which utilizes the performance function *g*, which is defined as zero at the limit state. The approach is based on assumptions that all input parameters are normally distributed, and that the limit state is also a normally distributed variable. FOSM and FORM cannot be used with different types of variable distributions. Also, the two approaches usually provide some ‘first order’ approximations. The Monte Carlo simulation method utilizes random number simulation to extrapolate probability density function values (Baecher and Christian, 2003; Harr, 1987). The inputs for a simulation process for a variable are its mean value, either standard deviation or coefficient of variation (COV), as well as its type of distribution. Any input can be set as a probabilistic variable if its mean value, standard of deviation or COV, and the distribution function type are provided. According to Baecher and Christian (2003), the Monte Carlo technique has the advantage because it is relatively easy to implement on a computer and can deal with a wide range of functions. The major disadvantage is that the results may converge very slowly. As stated by Allen et al. (2005), when “a closed-form solution is either not available or is considered too approximate, Monte Carlo simulation can be performed.” The Monte Carlo simulation method is more flexible and rigorous, and if enough simulations are generated, the results approach exact solutions; thus, the Monte-Carlo simulation method was used in this research for probabilistic analyses.

*Shaft head displacement calculation using the t-z method:* The load transfer method, or *t-z* method, is often used to calculate shaft head displacement (O'Neil and Reese, 1999; Misra and Roberts, 2009; Brown et al., 2010). The method requires predictive models for ultimate unit side and tip resistance well as load transfer models to predict mobilization of resistance along the shaft. Models for the ultimate unit side and tip resistance (Eqs. (2) and (3)) were developed from a large collection of load test measurements for full-scale drilled shafts founded in shale throughout the state of Missouri (Loehr et al., 2011).

(2) $$q_{s}=1.71\times UCS^{0.79\;}\;\leq \;\;1,436\;\text{kPa}\;$$

(3) $$\;q_{p}=43.0\times UCS^{0.71}\;\;\leq \;\;19152\;\text{kPa}$$

where $q_{s}$ is the ultimate unit side resistance and $q_{p}$ is the ultimate unit tip resistance. The variability and uncertainty associated with these models were quantified by a coefficient of variation of 0.66 and 0.25, respectively. Load transfer models were developed from measurements for a large collection of full-scale load tests on shales in the states Missouri, Kansas, Colorado (Vu, 2013). Models for the unit side and tip resistance (Eqs. (4) and (5)) drawn from this work are:

(4) $$t=\frac{z}{az+b}=\frac{z}{1.07*z+0.13}$$

(5) $$q=\frac{w}{aw+b}=\frac{w}{1.1*w+0.72}$$

where t and q are normalized unit side and tip resistance, respectively; z and w are normalized displacement along the shaft side and tip, respectively; and a and b are fitting parameters derived from the load test measurements. The standard deviation of the t*-*z model is 0.17, and the q*-*w is 0.14 (Vu, 2013).

## 3 Methods

The factored strength approach (Becker, 1996; Salgado, 2008) in which the geomaterial strength is factored and was used in this research because it offers greater flexibility and a potential for greater precision due to the resistance factors, which are easily related to the variability and uncertainty present in relevant design parameters (Becker, 1996, Vu and Loehr, 2015, 2017). Most design methods for drilled shafts in shale/rock are based on the uniaxial compressive strength (UCS). The SLS resistance factor, φ, is therefore applied to UCS to account for the uncertainty present in a design. The factored uniaxial compressive strength, $UCS^{*}$, is calculated as

(6) $$UCS^{*}=\varphi *UCS$$

Then $UCS^{*}$ is used as an input for the *t-z* method to determine factored shaft head displacement, $y^{*}$, in the same manner as the traditional approach of using *UCS* to determine shaft head displacement *y*. The SLS design check is then based on the requirement that the factored displacement, $y^{*}$, be less than some established allowable or limiting settlement, $y_{a}$ where the SLS is enforced by the criterion (Vu and Loehr, 2017):

(7) $$y_{a}-y^{*}\;\geq 0$$

SLS resistance factors were calibrated using a computer program written in MATLAB^®^ to implement load transfer analyses using the finite element method and the Monte Carlo simulation technique (Vu, 2013; Vu and Loehr, 2017). The program computes the top of foundation's vertical displacement under a given probabilistic load based on the following proposed procedure:

1. Generate probabilistic values for dead load (DL), live load (LL), shaft stiffness (EA), material strength (UCS), and ultimate unit side and tip resistance ($q_{s}$ and $q_{p}$, respectively) according to specified distributions of the parameters. Randomly generated values of *EA*, *UCS* and *q*_*s*_ are different for different element of the shaft;
2. Generate probabilistic load transfer (i.e. t*-*z and q*-*w) functions for each element according to the variability and uncertainty associated with the load transfer functions;
3. Determine the foundation displacement for each set of probabilistic parameter values;
4. Establish the number of “SLS failure cases”, $n_{f}$, associated with the predetermined target probability of failure $p_{f}$;
5. Determine the factored displacement, $y^{*}$ corresponding to the number of SLS failure cases, $n_{f}$ by sorting the computed displacements in descending order and taking the ${\left(n_{f}+1\right)}^{th}$ displacement value as $y^{*}$;
6. Calculate the factored uniaxial compressive strength $\left(UCS^{*}\right)$, that produces $y^{*}$ by computing vertical displacement while reducing the values of UCS until the computed displacement is equal to the value of $y^{*}$. Other parameters were set to their mean values;
7. Compute the SLS resistance factor as in Eq. (8):

(8) $$\varphi =\frac{UCS^{*}}{UCS}$$

The procedure was developed so that if a design uses a factored $UCS^{*}$ and satisfies Eq. (7), the design will achieve a predetermined target probability of failure.

*Input Parameters for Probabilistic Analysis of SLS:* For an SLS design based on the *t-z* method, there are a total of 11 deterministic and probabilistic variables, resulting in a total of 24 inputs, not including type of probabilistic distribution. The shaft length and the probability of failure are the only two variables that are considered deterministic. All 24 inputs are listed below:

a) Geomaterial strength and its variability/uncertainty, represented by its coefficient of variation (two inputs);
b) Dead load and its variability/uncertainty (two inputs);
c) Live load and its variability/uncertainty (two inputs);
d) Shaft length, considered deterministic (one input);
e) Shaft diameter and its variability/uncertainty (two inputs);
f) Concrete Young's modulus and its variability/uncertainty (two inputs);
g) Probability of failure, considered deterministic (one input);
h) *t-z* and *q-w* fitting parameters (four inputs for two pairs of fitting parameters) and their standard deviations (two inputs), and;
i) Ultimate unit side resistance (two inputs for two parameters), ultimate unit tip resistance (two inputs) and their coefficients of variation (two inputs).

**Fig. 2:**
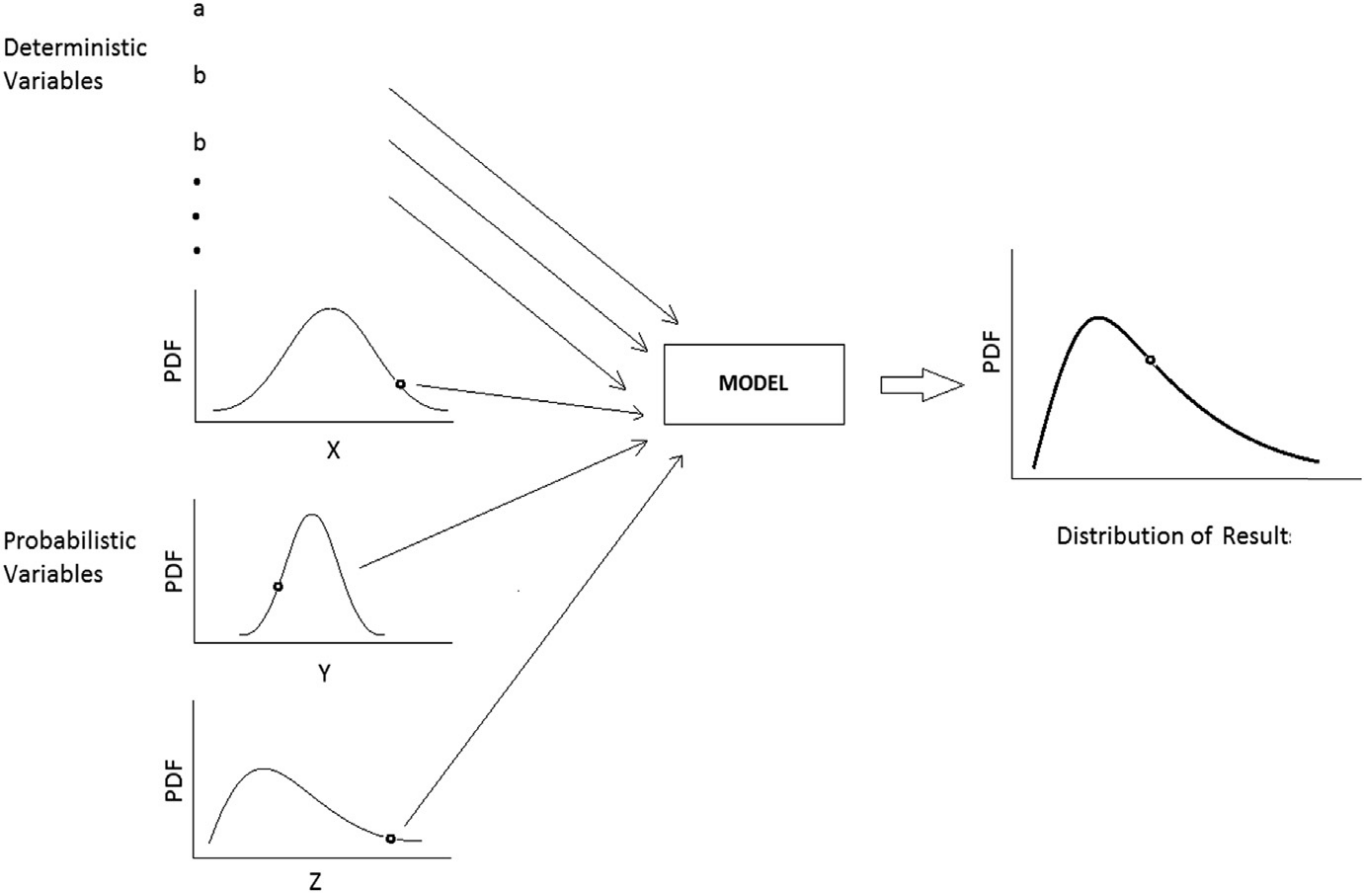
Schematic of Monte Carlo simulation.

*Monte Carlo Simulation:* Inputs for a Monte Carlo simulation of a variable include the variable's mean value, coefficient of variation (COV) or standard deviation, and its distribution type. Monte Carlo simulation uses random number simulations to establish the probability density function of parameter values for every probabilistic variable (Fig. 2). All 24 listed inputs were used to generate the probability density function of the output. For one simulation, one value is taken from the probability density function of each probabilistic parameter, and then, along with the other deterministic variable values, all values are put into a load-transfer model to calculate one value of shaft head vertical displacement. The process is repeated for *n* simulations to obtain the shaft head displacement probability density function.

**Fig. 3:**
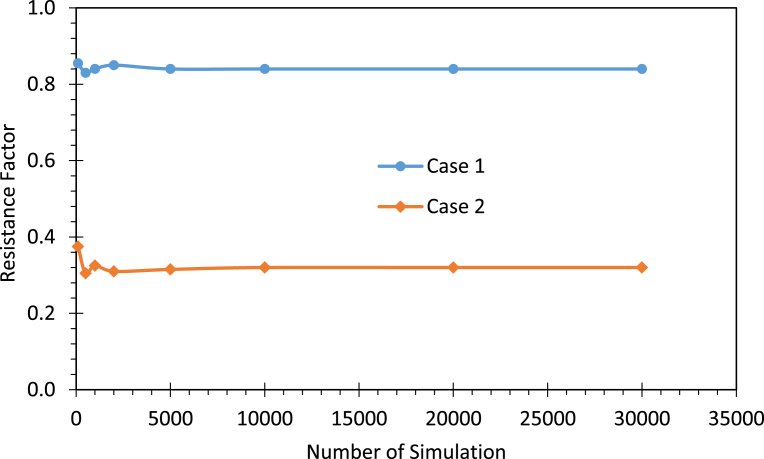
Resistance factor versus number of Monte Carlo simulations.

Since Monte Carlo simulation method is an approximate method, its accuracy is largely dictated by the number of simulations *n* that are performed. Allen et al. (2005) stated that 5,000 to 10,000 simulations or more are needed to adequately define the distribution of the limit state function for a probability index of β_T_ also = 2.3 to 3.0, which is greater than is usually required for SLS. Harr (1987) used the binominal distribution function and reliability theory to show that if it is desired that “the Monte Carlo simulation not to differ by more than 1% from the estimated value with 99% confidence”, 16,641 trials would be required. Two examples were set up to determine an SLS resistance factor, which is an indirect measure related to the reliability or probability of failure (Vu, 2013), with the resulting resistance factors are plotted in Fig. 3. The resistance factors are almost identical when the number of simulations exceeds 5000. In this research, the number of simulations was chosen to be 30,000.

**Fig. 4:**
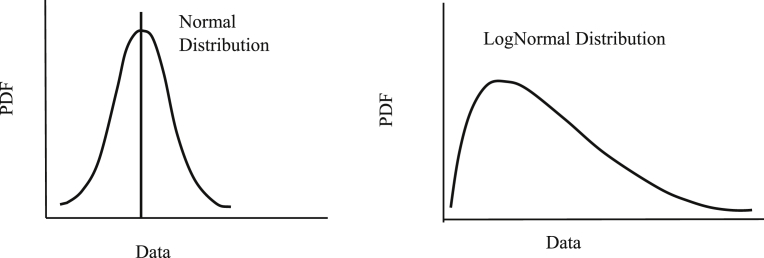
Normal (left) and lognormal distributions (right).

*Random number generation of variables*: In geotechnical engineering, the most frequently used distribution types for probabilistic variables are the normal and log-normal distributions (Phoon et al., 1995; Duncan, 2000; Baecher and Christian, 2003; Allen et al., 2005). The appeal of the normal distribution is that it is mathematically convenient; it accurately reflects many measurements, and it is commonly used in practice. The normal distribution is bell-shaped (Fig. 4). However, the normal distribution often includes some negative values, which are impractical and unacceptable for many SLS design problems. The log-normal distribution type reflects data where the natural logarithms of the data are normally distributed. The shape of the distribution is an eccentric bell with a much longer tail (Fig. 4). This type of distribution is strictly non-negative and is used more often. In this research, the types of distributions for the input variables are chosen based on field test data, or taken from well-established literature.

*Method to Generate Normally Distributed Parameter Values:* If the meanμ, standard deviationσ, and distribution type of a parameter are known, the Monte Carlo approach can simulate *n* numbers of random parameter values that have the same mean, standard deviation and distribution type. In MATLAB^®^, for a variable that is normally distributed with mean μ and standard deviation σ*,* a random parameter value set from *n* simulations can be produced using the Eq. (9):

(9) X=μ+σ∗randn(1,n)

where *randn* is a command to generate an array of *n* random numbers that have standard normal distribution with a mean of zero and standard deviation of unity.

If the data are highly variable and the standard deviation *σ* is large, it is possible for the process to produce negative values that are non-real. Generated negative values are replaced with positive, near-zero values (10^−6^ for all cases). This approach is more beneficial than to assume a lognormal distribution even though the data show normal distribution characteristics, which is the common practice found in literature.

*Method to Generate Generation of Lognormally Distributed Parameter Values:* In order to generate a set of a variables *L*, which are lognormally distributed with mean μ and standard deviation σ*,* a transformation step must be performed (Eqs. (10), (11), (12), and (13)). The logarithm of the variable *L* is N=ln(L).*N* is a normally a distributed variable with mean *λ* and standard deviationξ. The relationships between the mean *λ* and standard deviationξ of variables *L* and mean μ and standard deviation σ of the normally distributed variable *N* are:

(10) $$\xi =\sqrt{\text{ln}\left(1+\frac{\sigma^{2}}{\mu^{2}}\right)}$$

(11) $$\lambda =ln\mu -\frac{\xi^{2}}{2}$$

The variable *N* can be generated using following function:

(12) N=λ+ξ∗randn(1,n)

The final step is to obtain the data set L is by taking the exponential of the values in *N:*

(13) $$L=e^{N}$$

**Fig. 5:**
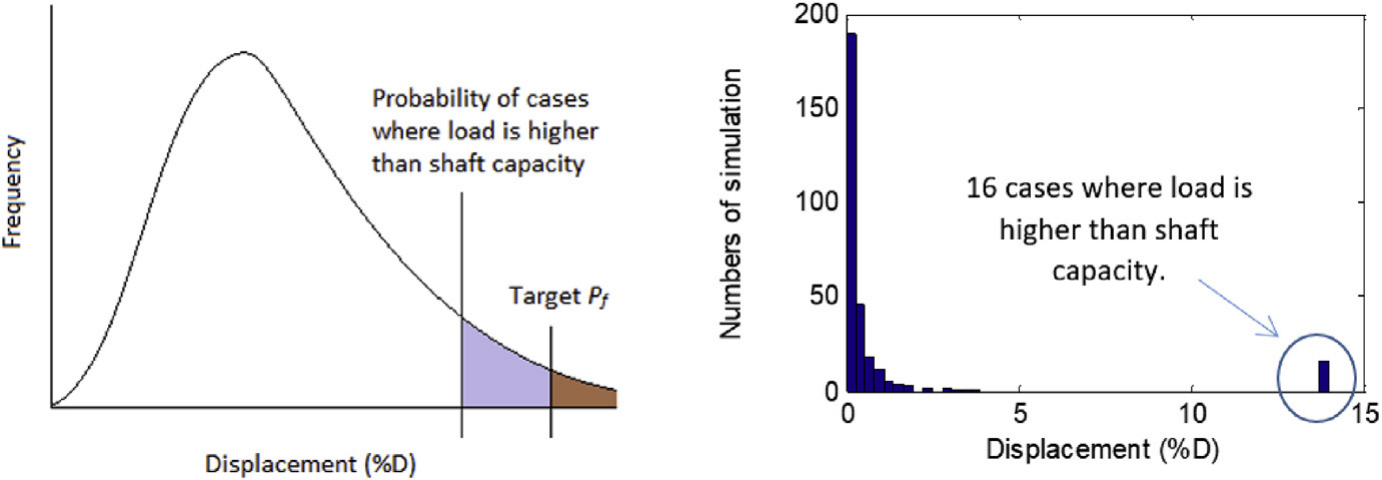
Impossible case: the probability of failure cases is larger than the target probability of failure (left), and in the displacement histogram from 300 simulations (right).

*Possible and Impossible Case:* Cases have been reported wherein randomly simulated loads were higher than the randomly simulated shaft resistances (or shaft capacities). When this happens, the shaft head displacement for these cases cannot be calculated. If the number of these cases is larger than the target probability of failure, as illustrated in Fig. 5, no resistance factor can be obtained to achieve the SLS target probability of failure. This situation is called the *“impossible”* case. The low shaft resistance comes from a combination of resistance components, such as small *t* value (from *t-z* model) or small UCS.To illustrate this concept, an example of 300 Monte Carlo simulations was run to obtain a histogram of resulting shaft displacements in a percentage of the shaft diameter (%D). For a simulation when the randomly generated load was higher than the randomly generated capacity, the solution for that simulation did not converge, and the displacement was assigned an arbitrary large displacement, i.e., 14% of the diameter. Out of 300 simulations, there were 16 simulations where loads were higher than shaft capacity as in Fig. 5 (right). If the target *P*_*f*_ was of 1/100 (<16/300), then the resistance factor cannot be determined; however, if the target *P*_*f*_ is 1/15 (>16/300) then the resistance factor can be obtained. This research uses normalized load θ, which is the ratio of load (sum of dead load and live load) and the nominal shaft capacity (Vu and Loehr, 2017). When the normalized load is high, and uncertainty and variability of the resistance of soil/shale properties also are high, the load distribution and the resistance distribution “move” closer together (Fig. 1), the resistance distribution becomes wider, and the overlap area becomes larger. This means that the failure cases are more likely to occur.

In an SLS design, if the designed shaft has conditions of possible case, three different ways exist whereby conditions can be moved into the impossible case. The designer could increase the shaft length or diameter, so the normalized load θ is reduced where technically the resistance distribution is shifted farther from the load distribution. Theoretically, the designer can change the COVofUCS by conducting more site exploration tests, or the designer can increase the target probability of failure *P*_*f*_, although this is not practical.

**Fig. 6:**
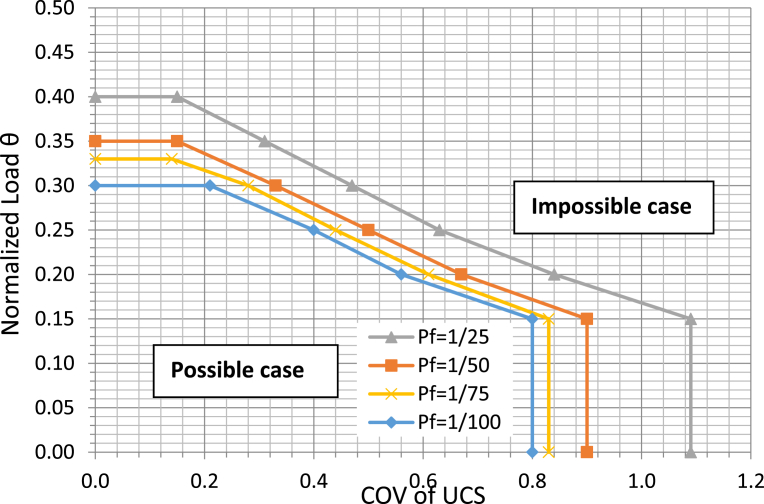
Boundaries of possible and impossible cases for L/D of 10.

The impossible case for a certain normalized shaft length is formed by a combination of normalized load *θ*, COV of *UCS* and the target probability of failure *P*_*f*_. The boundary of the case was found by making the number of the impossible cases equal to the SLS probability of failure. The case boundaries for a normalized shaft length L/D of 10.0 are presented in Fig. 6 and Table 1. As shown in Fig. 6, four curves are associated with four target probabilities of failure. The *left-and-under* area of each curve is a possible case area, while the *right-and-above* area is the impossible case area. Here, the target probability of failure cannot be achieved no matter how small the resistance factor is, and the case is unfavorable for a design.

**Table 1:** Combinations for impossible case with L/D of 10.

Normalized Load	COV of *UCS*			
	*P*_*f*_ = 1/25	*P*_*f*_ = 1/50	*P*_*f*_ = 1/75	*P*_*f*_ = 1/100
0.15	1.09	0.90	0.83	0.80
0.20	0.84	0.67	0.61	0.56
0.25	0.63	0.50	0.44	0.40
0.30	0.47	0.33	0.28	0.21
0.35	0.31	0.15		
0.40	0.15		Impossible	

## 4 Results & discussion

**Fig. 7:**
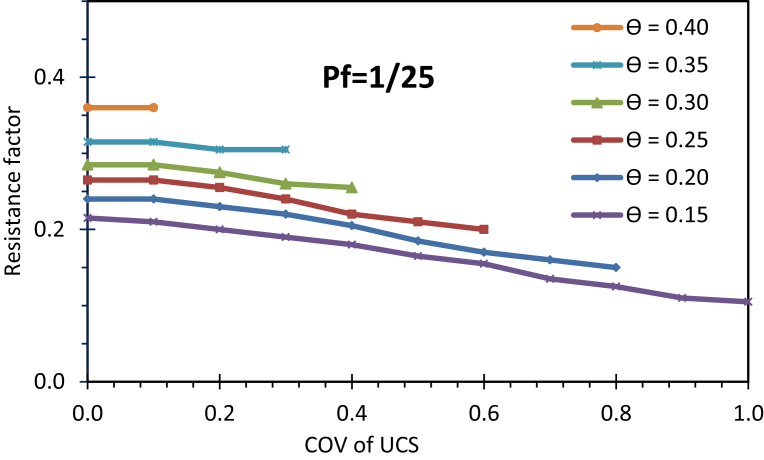
Resistance factors for different normalized loads for L/D = 10.

Resistance factors were calibrated for drilled shafts at SLS at different *P*_*f*_, L/D, normalized load, and COV of *UCS*. The inputs and sources are presented in Table 2, only shaft diameter and length are considered deterministic. Fig. 7 presents resistance factors for *P*_*f*_ = 1/25, *L/D* = 10. More resistance factors can be found at Vu and Loehr (2017). The resistance factors appear to be low, ranging from 0.10 to 0.36. High variability in the load transfer models and predictive models, together with accounting for more sources of uncertainty in the calibration process, can explain the lower values of these SLS resistance factors for individual drilled shafts in shale. However, an SLS resistance factor that is calibrated while accounting for fewer probabilistic parameters will produce an unconservative design.

**Table 2:** Inputs for calibration of SLS resistance factors.

Parameters	Nominal Values	COV	Standard Deviation	Source
Dead load, DL (kN)	Varied to produce desired θ	0.10	-	Kulicki et al. (2007)
Live load, LL (kN)	DL/2	0.12	-	Kulicki et al. (2007)
Material strength, UCS (kPa)	383	0.0 to 1.0	-	-
Shaft diameter, D (m)	0.9	Deterministic	-	-
Shaft length, L (m)	Varied to produce desired L/D	Deterministic	-	-
Concrete modulus, E (MPa)	28200	0.15	-	Tyler (2010)
Target probability of failure, $p_{f}$	1/25, 1/50, 1/75, and 1/100	-	-	Huaco et al. (2012)
Mobilized side resistance, t	$\frac{t}{q_{s}}=\frac{z}{1.07\cdot z+0.13}$	-	0.17	Vu (2013)
Mobilized tip resistance, q	$\frac{q}{q_{p}}=\frac{w}{1.10\cdot w+0.72}$	-	0.14	Vu (2013)
Ultimate unit side resistance, $q_{s}$	$q_{s}=1.71\cdot UCS^{0.79}$	0.66	-	Vu and Loehr (2015)
Ultimate unit tip resistance, $q_{p}$	$q_{p}=43\cdot UCS^{0.71}$	0.25	-	Vu and Loehr (2015)

**Fig. 8:**
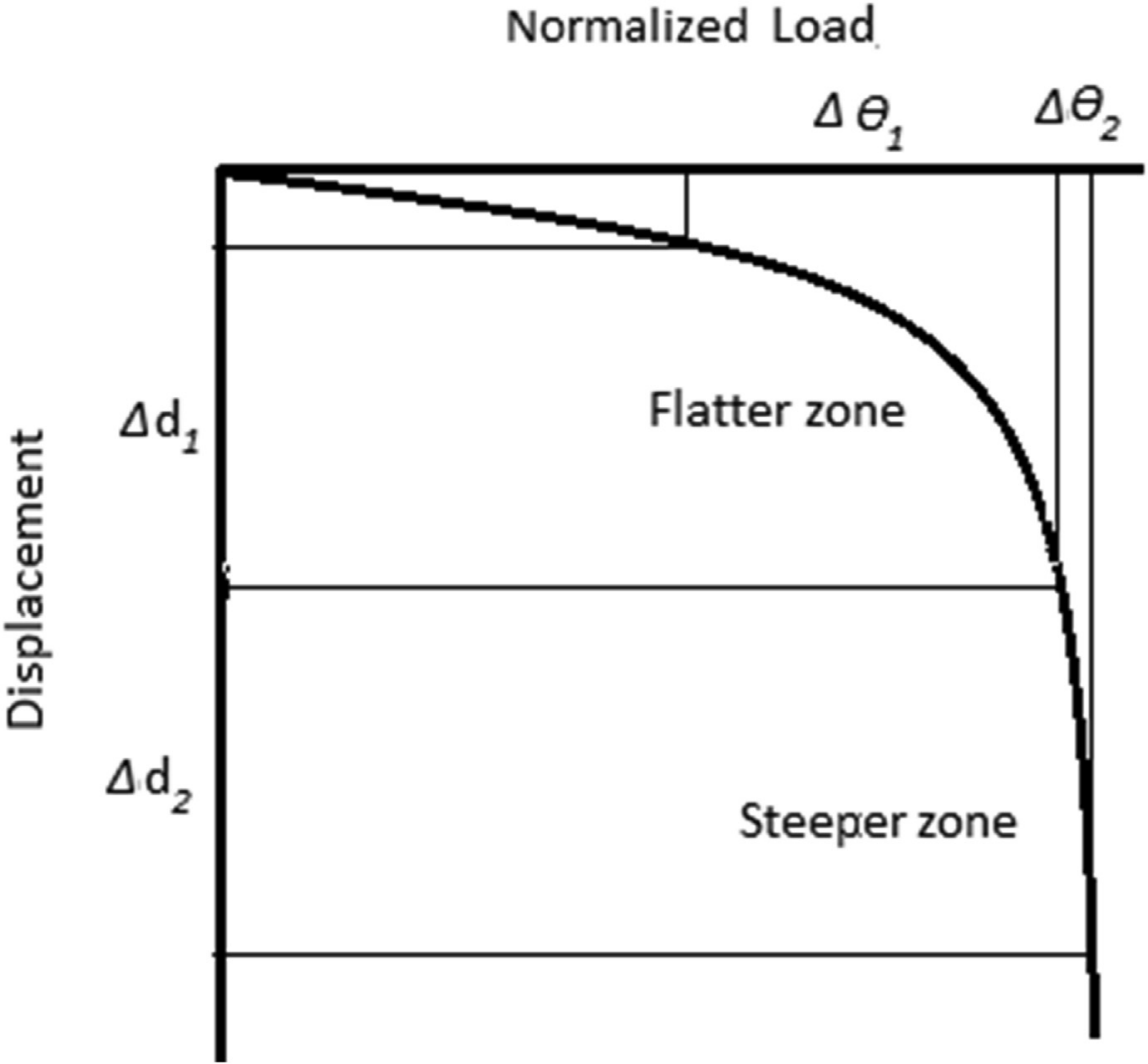
Normalized load versus shaft displacement.

As observed in this study, the resistance factor is *dependent* on load: the higher the normalized load is, the lower the resistance factor is. At a COVofUCS equal to zero, the resistance factor significantly decreases from 0.36 to 0.21 when the normalized load θ varies from 0.40 to 0.15. The curve for a higher normalized load of 0.4 truncates when COVofUCS is 0.1, meaning that the target probability of failure, which is 1/25, cannot be achieved when the COVofUCS is higher than 0.1 (which is attributed to the impossible case). For a lower load of 0.15, the target *P*_*f*_ can be reached even when the COVofUCS is as high as 1.0. With the lower normalized load, a design is more likely to achieve the target probability of failure with the higher COVofUCS, and the impossible case is less likely to occur. Fig. 8 can be used to qualitatively explain how the resistance factor is dependent of load, as it displays a highly nonlinear relationship of normalized load versus displacement. The effect of changing the load is *inversely* proportional to the effect of changing the *UCS*. As for the strength factor approach, the resistance factor is used to factor or reduce *UCS* to increase the nominal value of the settlement to the factored settlement y* that is associated with the target probability of failure. The effect of reducing the *UCS* is similar to the effect of increasing the load. As in Fig. 8, to obtain the same increasing amount of displacement $\Delta d=\Delta d_{1}\;=\Delta d_{2}$, the required change in the normalized load $\Delta \theta_{1}\;$ in the flatter zone is much larger than the $\Delta \theta_{2}\;$ in the steepter zone. This means that less change in normalized load is required in the flatter zone. The reduced change in normalized load is analogous to less change in *UCS* (recall they are inversely proportional), and the less change in *UCS* means a higher resistance factor is needed to obtain the factored displacement y*, or the resistance factor is higher for the higher normalized load. Since the resistance factor was determined to be a function of normalized load, the design of SLS for drilled shafts becomes a cumbersome process, meaning the engineers need to obtain different resistance factors for different loading or nominal shaft capacity which in turn depends in shaft dimensions. This is possibly the reason why Zhang and Chu (2009) proposed SLS resistance factors strictly only for use with nominal working loads equal to 50 percent of the ultimate foundation capacity, and the resistance factors by Misra and Roberts (2009) were proposed only for specific foundation dimensions. The design procedure proposed presented below overcomes this cumbersomeness and provides a flexibility in design of drilled shaft foundation at SLS.

The results from resistance factor calibration can be used in the following procedure for the design of drilled shafts in shale at the SLS. The procedure is flexible and easy to use, and contains the following five steps:

1. Obtain initial shaft dimensions using strength limit state criteria. From the dimensions, calculate the nominal shaft capacity, $R_{n}$.
2. Determine normalized load, θ based on the factored load for the SLS.
3. Obtain resistance factor φ for the given COV of UCS (Fig. 7).
4. Compute factored shaft head vertical displacement, $y^{*}$using t-z method (can use any software tools e.g., in-house computer codes or commercial software that can model the load transfer response represented by Eqs. (2), (3), (4), and (5) as inputs).
5. Compare $y^{*}$ to the established allowable settlement,$\;y_{a}$. If the design requirement (Eq. (7)) is not met then repeat Steps 1 to 5 for increasing drilled shaft dimensions (mostly shaft length) until the design requirement is met.

## 5 Example

**Fig. 9:**
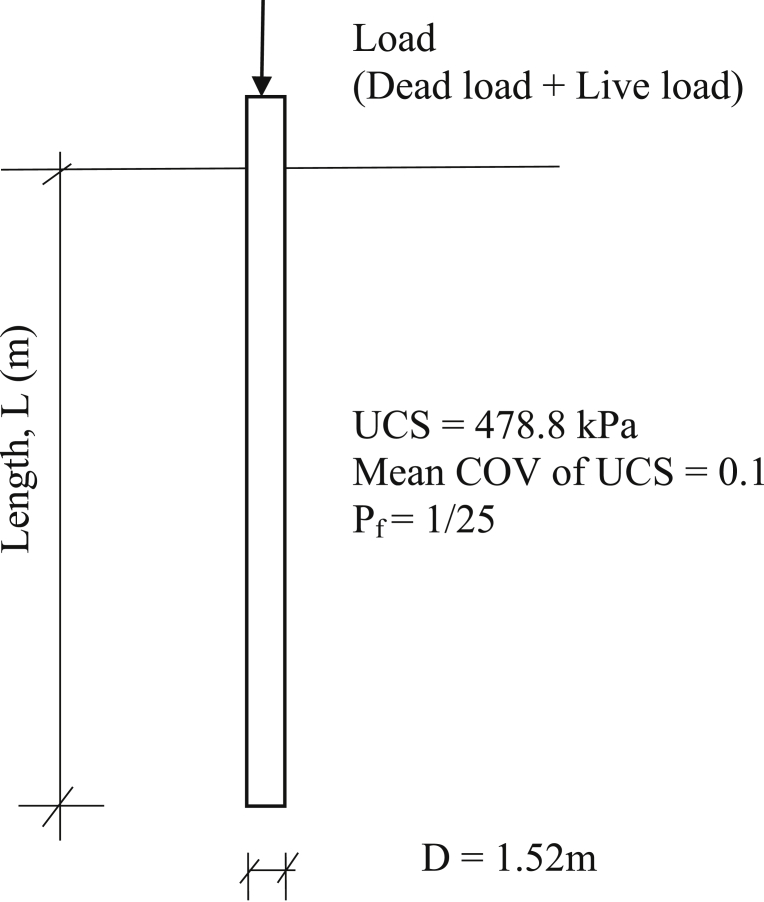
Example to demonstrate the design procedure.

A drilled shaft is founded in shale with a mean UCS of 500 kPa, coefficient of variation is COV is 0.1 (Fig. 9). Dead load (DL) is 3780 kN and live load (LL) is 1890 kN. The allowable displacement$\;y_{a}$, is 15 mm, and the target probability of failure is 1/25. The problem is solved following the 5-step procedure:

1) Use strength limit state requirements to determine initial shaft dimensions: shaft diameter of D = 1.52 m and shaft length of L = 15.2 m. The nominal shaft resistance, $R_{n}$, is then calculated as 22,900 kN.
2) Calculate normalized load:
  $$\theta =\;\frac{DL+LL+\;W_{s}\;}{R_{n}}=\frac{\left(3780+1890+\;655\right)}{22.9\;x\;10^{3}\;}=0.276.$$
3) With L/D = 15.2/1.52 = 10, and the given $p_{f}$ = 1/25, the resistance factorφ=0.27 is then obtained from Fig. 7.
$\;UCS^{*}$ is calculated using Eq. (6):
  $$UCS^{*}=\varphi *UCS=\;0.27*500=133.5\;\text{kPa}$$
4 Applying $UCS^{*}=$133.5 kPa and the *t-z* models as in Eqs. (2), (3), (4), and (5), employ *t-z* method to determine the factored settlement $y^{*}$ of 16.8 mm.
5) Because the factored shaft displacement of 16.8 mm is greater than 15 mm the allowable displacement, the shaft length is increased. Steps 1 to 5 are repeated with the new increased shaft length. After several trial and errors, a shaft length of L=16.1 m yields a factored shaft head displacement of 14.9 mm, less than the allowable settlement of 15 mm. Shaft dimensions of *D* = 1.5 m and *L* = 16.1 m are chosen for the design.

## 6 Conclusions

Monte Carlo simulation method for probabilistic analyses and for calibration of resistance factors for drilled shafts at SLS is introduced. Recommendations and observations were made advocating random number generation using Monte-Carlos simulations. A discussions on the finding of an impossible case in which resistance factors cannot be calculated in some circumstances is presented. Resistance factors for drilled shafts in shale are introduced, and were found to be responsive to normalized load, and the higher the normalized load, the lower the resistance factor.

## Declarations

### Author contribution statement

Thuy Vu: Conceived and designed the experiments; Analyzed and interpreted the data; Wrote the paper.

Erik Loehr: Performed the experiments.

Douglas Smith: Contributed reagents, materials, analysis tools or data.

### Funding statement

This research did not receive any specific grant from funding agencies in the public, commercial, or not-for-profit sectors.

### Competing interest statement

The authors declare no conflict of interest.

### Additional information

No additional information is available for this paper.

## References

[bib1] AASHTO (2014). LRFD Bridge Design Specifications.

[bib2] Allen T.M., Nowak A.S., Bathurst R.J. (2005). Calibration to Determine Load and Resistance Factors for Geotechnical and Structural Design.

[bib3] Ang A.H.S., Tang W.H. (2004). Probability concepts in engineering. Planning.

[bib4] Baecher G., Christian J. (2003). Reliability and Statistics in Geotechnical Engineering.

[bib5] Becker D.E. (1996). Eighteenth Canadian Geotechnical Colloquium: limit states design for foundations. Part I. An overview of the foundation design process. Can. Geotech. J..

[bib6] Brown D.A., Turner J.P., Castelli R.J. (2010). Drilled Shafts: Construction Procedures and LRFD Design Methods.

[bib7] Duncan J.M. (2000). Factors of safety and reliability in geotechnical engineering. J. Geotech. Geoenviron. Eng..

[bib8] Griffiths D.V., Fenton G.A. (2007).

[bib9] Harr M.E. (1987). Reliability based design in civil engineering (reprint 1996 of the original edition 1987) paper. Recherche.

[bib10] Huaco D.R., Bowders J.J., Loehr J.E. (2012). Method to develop target levels of reliability for design using LRFD. Paper Presented at the Transportation Research Board 91st Annual Meeting.

[bib11] Kulicki J.M., Prucz Z., Clancy C.M., Mertz D.R., Nowak A.S. (2007). Updating the Calibration Report for AASHTO LRFD Code.

[bib12] Loehr J.E., Bowders J.J., Ge L., Likos W.J., Luna R., Maerz N., Stephenson R.W. (2011). Engineering Policy Guidelines for Design of Drilled Shafts.

[bib13] Misra A., Roberts L.A. (2009). Service limit state resistance factors for drilled shafts. Geotechnique.

[bib14] O'Neil M.W., Reese L.C. (1999). Drilled Shafts: Construction Procedures and Design Methods.

[bib15] Phoon K.K., Kulhawy F.H., Grigoriu M.D. (1995). Reliability-based Design of Foundations for Transmission Line Structures.

[bib16] Phoon K.-K., Kulhawy F.H., Grigoriu M.D. (2003). Development of a reliability-based design framework for transmission line structure foundations. J. Geotech. Geoenviron. Eng..

[bib17] Phoon K.K., Kulhawy F.H. (2008). Serviceability limit state reliability-based design. Reliability-based Design in Geotechnical Engineering–Computations and Applications.

[bib18] Salgado R. (2008). The Engineering of Foundations.

[bib19] Tyler H.L. (2010). Influence of Parameter Variability on Side Shear Values Determined from O-cell Testing of Drilled Shafts.

[bib20] Vu T. (2013). Load and Resistance Factor Design of Drilled Shafts at the Service Limit State.

[bib21] Vu T., Loehr E. (2015). Service Limit State Design for Individual Drilled Shafts in Shale Using T-z Method.

[bib22] Vu T., Loehr E. (2017). Service limit state design for individual drilled shafts in shales. J. Geotech. Geoenviron. Eng..

[bib23] Zhang L., Chu F. (2009). Developing partial factors for serviceability limit state design of large-diameter bored piles. Int. Foundations Congress and Equipment Expo ‘09 (IFCEE09), Orlando, FL.

